# A comparative cross-sectional study of some hematological parameters of hypertensive and normotensive individuals at the university of Gondar hospital, Northwest Ethiopia

**DOI:** 10.1186/s12878-017-0093-9

**Published:** 2017-11-28

**Authors:** Bamlaku Enawgaw, Nigist Adane, Betelihem Terefe, Fikir Asrie, Mulugeta Melku

**Affiliations:** Department of Hematology & Immunohematology, School of Biomedical and Laboratory Sciences, College of Medicine and Health Sciences, University of Gondar, Gondar, Ethiopia

**Keywords:** Hypertension, Hematological parameters, Blood pressure indices, Gondar, Ethiopia

## Abstract

**Background:**

Hypertension is a major health problem worldwide. It can lead to cardiovascular disease and also leads to functional disturbances including hematological parameters. The abnormalities of haematological parameters may enhance an end-organ damage. Therefore, the aim of this study was to assess some hematological parameters of hypertensive individuals in comparison with normotensive individuals at University of Gondar hospital, northwest Ethiopia.

**Methods:**

A cross sectional comparative study was conducted from October to November 2015 on a total of 126 hypertensive and 126 normotensive individuals at University of Gondar Hospital. All participants after taking informed consent were interviewed for detailed history and 3 ml of blood was collected for hematological test analysis. Independent *t*-test and the Mann Whitney *u*-test were used to find out significant difference and Pearson’s and Spearman’s correlation were used for correlation test. *P* values less than 0.05 was considered the level of significance.

**Result:**

From a total of 252 study subjects, about 67.5% were females. The mean age of study subjects was 50.3 ± 11 years for hypertensive individuals and 49.8 ± 11.6 years for normotensive individuals with range of 18–65 years. In the present study, the median (IQR) value of WBC, RBC, Hgb, HCT, MCV and the mean value of MCHC, RDW, MPV and PDW were significantly higher in hypertensive group compared to apparently healthy normotensive groups. Additionally, WBC, RBC, Hgb, HCT and PLT showed statistically significant positive correlations with blood pressure indices. Platelet count and MCH did not show statistically significant difference between the two groups.

**Conclusion:**

Hypertension has impact on hematological parameters. In this study, the mean and median values of haematological parameters in hypertensive individuals were significantly different compared to apparently healthy normotensive individuals. Hence, hematological parameters can be used to monitor the prognosis of the disease and manage hypertensive related complications, and it is important to assess hematological parameters for hypertensive individuals which may help to prevent complications associated hematological disorders.

**Electronic supplementary material:**

The online version of this article (doi: 10.1186/s12878-017-0093-9) contains supplementary material, which is available to authorized users.

## Background

Hypertension (HTN) is also called high blood pressure. It is a condition in which systemic arterial pressure is elevated above the threshold value [1]. It is expressed by systolic (maximum) and diastolic (minimum) arterial pressures. Systolic pressure is occurring during contraction of the left ventricle of the heart while diastolic pressure is occurring before the next contraction. Normally at rest the systolic pressure is within 100–140 mm mercury (mmHg) and diastolic pressure is within 60–90 mmHg [2, 3]. Based on the seventh Joint National Committee (7 JNC) report in 2008, normal HTN was defined as systolic blood pressure (SBP) < 120 mmHg and diastolic blood pressure (DBP) < 80 mmHg, pre-HTN with SBP of 120-139 mmHg or DBP 80–89 mmHg, stage I HTN with SBP of 140–159 mmHg or DBP 90–99 mmHg and stage II HTN with SBP ≥ 160 mmHg or DBP ≥ 100 mmHg [1, 4].

HTN can be categorized in to two; primary and secondary hypertensions. Primary HTN, which consists of about 95% cases, can occur without any obvious underlying causes while secondary HTN is developed due to secondary to diseases such as kidney disease, endocrine disorders and narrowing of the aorta or kidney arteries [5, 6].

HTN is a major health problem worldwide that affects 20–30% of the adult population [7]. It is rapidly increased from 3% in rural areas to 30% in urban areas of Sub-Saharan Africa. In 2008, its overall prevalence rate in Sub Saharan Africa was 16.2% ranging from 10.6% (Ethiopia) to 26.9% (Ghana) [8–10].

HTN may lead to severe end-organ damage, coronary heart disease and stroke which constitute the leading cause of mortality [11, 12]. It is strongly associated with functional and structural abnormalities to organs that involve in hematopoiesis [5, 6, 13] and blood viscosity is increased in most hypertensive patient’s [14]. Although, the details of this association is unclear, development of HTN is accompanied by reduction in deformability, and an increase in size, number and aggregability of red blood cells. These abnormalities of the red cells may worsen the microcirculation and enhance an end-organ damage [11, 15].

On the other hand, HTN has an impact on hematological parameter such as hematocrit (HCT), hemoglobin (Hgb), red blood cell (RBC) count, white blood cell (WBC) count and platelet (PLT) count. Impaired hematological parameters may strongly indicate hypertensive end-organ damage, specifically kidney failure [7, 16, 17]. Specifically increased Hgb level may cause left ventricular hypertrophy while low Hgb levels causes anemia and heart failure [18].

Generally, there are contradictory results regarding hematological parameters of hypertensive patients in different countries. Moreover, there is lack of information regarding to hematological parameters in hypertensive patients in Ethiopia. Therefore, this study was aimed at assessing hematological parameters in hypertensive patients in comparison with apparently healthy individuals and correlating hematological parameters with blood pressure indices (systolic blood pressure, diastolic blood pressure and mean arterial pressure) at university of Gondar hospital, Northwest Ethiopia.

## Methods

### Study setting, design and population

A comparative cross-sectional study was conducted from October to November 2015 at university of Gondar hospital chronic illness clinic. The hospital is located in Gondar town which is 740 km from the capital of Ethiopia, Addis Ababa, in the Northwest of Ethiopia. Gondar University Hospital is the only referral hospital in North West Ethiopia serving a population of about 5 million. This hospital gives different types of service for the population including treatment of chronic diseases. A total of 252 (126 hypertensive confirmed and 126 apparently healthy) study subjects were included conveniently in this study. The study subjects with hypertensive and healthy controls were age and sex matched. Study subjects with history of infectious diseases, alcohol consumers, smokers, taking antibiotic, treatment for anemia, patients with systemic diseases and with secondary hypertension were excluded from this study with face to face interview and review of medical records (Additional file 1).

### Data collection

#### Socio-demographic and clinical data collection

After taking a written informed consent data were collected by using structured questionnaire and by reviewing study subjects’ medical record (Additional file 1). The questionnaire was validated with pre-test.The data regarding anthropometric variables such as height (to the nearest centimeter without shoes) and weight (to the nearest 0.1 kg) were collected and body mass index (BMI) was calculated as weight in kilograms divided by height in meter squared. Blood pressure (BP) was measured by qualified personnel using an analog sphygmomanometer and stethoscope. All study subjects were recruited during their respective appointment schedule for follow up and newly diagnosed individuals. After interview and detailed review of the medical record, the study subjects were sent to laboratory where blood was collected for determination of complete blood cell count.

#### Laboratory sample collection and analysis

Laboratory analysis was done at University of Gondar hospital Laboratory. Three milliliters of venous blood were collected by experienced laboratory technologist from each study participants and complete blood cell count (CBC) was analyzed by using Sysmex KX-21 automated hematology analyzer. White blood cells (WBC), red blood cell parameters (RBC, Hgb, HCT, MCV, MCH, MCHC and RDW) and platelet parameters (PLT, MPV and PDW) were analyzed and collected for each study participants by using laboratory result registration form (Additional file 2).

### Quality assurance

The anthropometric and blood pressure measurements were taken twice and the average value was used. Protocol for sample collection, processing, and transportation was strictly followed to have safe procedure and reliable specimen. Quality controls and standard operating procedure were strictly followed for hematological parameter analysis. The results were properly documented, transcribed and reviewed.

### Statistical analysis

Data were cleaned, edited and checked for completeness and entered in to SPSS version 20 statistical software for analysis. The normality of data distribution was checked by statistical tools of Kolmogorov-Smirnov (K-S). The results were expressed as mean ± standard deviation and median with interquartile range. Comparison of parameters between hypertensive subjects and apparently healthy normotensive controls was done with independent *t*-test for normally distributed data and Mann-Whitney *U* test for non-normally distributed data. The correlation of hematological parameters with blood pressure indices (systolic blood pressure, diastolic blood pressure and mean arterial pressure) was assessed by Pearson’s and Spearman’s correlation. In any condition, *P* value < 0.05 was considered as statistically significant.

## Results

### Characteristics of study subjects

A total of 252 study subjects grouped into two groups (126 Hypertensive and 126 healthy controls) were involved in this study. From them 67.5% (85 hypertensive and 85 healthy) were female study subjects which makes 1:2 ratio of male to female. The mean age of study subjects was 50.3 ± 11 years for hypertensive individuals and 49.8 ± 11.6 years for apparently healthy individuals with range of 18–65 years. About 200 (79.4%) of the total study subjects and 84 (66.7%) of the cases group were urban dwellers. The mean and standard deviation value of the BMI, MAP, SBP and DBP were 24.0 ± 2.3, 109.4 ± 5.4 mmHg, 143.2 ± 8.0 mmHg, and 92.4 ± 4.6 mmHg, respectively. The average duration of illness was 3.47 ± 2.8 years (Table 1).

**Table 1:** Demographic and anthropometric characteristics of study participants at university of Gondar hospital from October to November 2015

	Variables	Hypertensive (*n* = 126)	Healthy controls (*n* = 126)
Age (years)	< 35 35–55 > 56	15 (51.7%) 65 (48.9%) 46 (51.1)	14 (48.3%) 68 (51.1%) 44 (48.9%)
Sex	Male	41 (32.5%)	41 (32.5%)
	Female	85 (67.5%)	85 (67.5%)
Residence	Urban	84 (66.7%)	116 (92.1%)
	Rural	42 (33.3%)	10 (7.9%)
BMI	< 18.5 18.5–24.9 25–29.9 > 30	1 (0.8%) 83 (65.9%) 40 (31.75%) 2 (1.6%)	1 (0.8%) 101 (80.2%) 24 (19.0%) 0 (0%)
Duration of illness (years)	< 5 5–10 > 10	101 (80.2%) 23 (18.3%) 2 (1.6%)	

### Comparison of hematological parameters

Hypertensive groups had significantly higher median (IQR) value of WBC, RBC, Hgb, HCT, MCV and mean (SD) value of MCHC, RDW, MPV and PDW were significantly higher in hypertensive patient than apparently healthy normotensive individuals (*P* < 0.05). Although the median (IQR) value of PLT count is relatively higher in hypertensive groups, there is no statistically significant difference between these groups (*P* = 0.262) (Table 2).

**Table 2:** Comparison of hematological parameters between hypertensive and normotensive groups at university of Gondar hospital from October to November 2015

Variables	Hypertensive (*n* = 126)		Healthy controls (*n* = 126)		*P* values
	Mean ± SD	Median (IQR)	Mean ± SD	Median (IQR)	
WBC (10^3^/μl)	–	6.90 (3.70)	–	5.20 (2.20)	< 0.001
RBC (10^6^/μl)	–	5.00 (0.88)	–	4.88 (0.58)	0.015
Hgb (g/dl)	–	14.60 (1.85)	–	14.15 (1.00)	0.005
HCT (%)	–	42.70 (5.80)	–	41.3 (3.00)	< 0.001
MCV (fl)	–	86.5 (6.13)	–	86.00 (4.43)	0.011
PLT (10^3^/^μ^l)	–	270.00 (101.00)	–	255.50 (103.25)	0.262
MCH (pg)	29.60 ± 1.75	–	29.61 ± 1.7	–	0.937
MCHC (g/dl)	34.27 ± 1.17	–	34.7 ± 1.13	–	0.003
RDW (SD) (fl)	43.19 ± 2.62	–	42.90 ± 4.42	–	< 0.001
MPV (fl)	10.0 ± 0.95	–	9.8 ± 1.0	–	0.046
PDW (fl)	12.6 ± 1.8	–	12.0 ± 1.7	–	0.007

**Note:** For hematological parameters expressed by median (IQR), *P* value is derived from Mann-Whitney *U* test; while those expressed by mean ± SD, *P* value is derived from independent T test

### Correlation of hematological parameters with blood pressure indices

In the present study correlation of the various haematological parameters and the blood pressure indices among hypertensive individuals was determined. As Table 3 indicates WBC, RBC, Hgb, HCT and PLT showed significant positive correlations with diastolic blood pressure, systolic blood pressure and mean arterial pressure (*r* = 0.282–3.96, *P* < 0.01).

**Table 3:** Correlation of haematological parameters with blood pressure indices among hypertensives individuals at university of Gondar hospital from October to November 2015

Variables	DBP (*P*-value)	SBP (*P*-value)	MAP (*P*-value)
WBC (10^3^/^μ^l)	0.362 (0.000) ^a^	0.396 (0.000) ^a^	0.371 (0.000) ^a^
RBC (10^6^/^μ^l)	0.358 (0.001) ^a^	0.303 (0.000) ^a^	0.301 (0.001) ^a^
Hgb (g/dl)	0.341 (0.000) ^a^	0.282 (0.001) ^a^	0.286 (0.001) ^a^
HCT (%)	0.368 (0.000) ^a^	0.284 (0.001) ^a^	0.300 (0.001) ^a^
MCV (fl)	−0.136 (0.130)	−0.147 (0.101)	−0.118 (0.189)
MCH (pg)	−0.088 (0.330)	−0.097 (0.282)	−0.074 (0.410)
MCHC (g/dl)	0.011 (0.904)	0.030 (0.742)	−0.003 (0.904)
RDW (SD) (fl)	−0.031 (0.734)	0.037 (0.685)	0.085 (0.346)
PLT (10^3^/^μ^l)	0.376 (0.000) ^a^	0.373 (0.000) ^a^	0.362 (0.000) ^a^
MPV (fl)	−0.149 (0.97)	−0.062 (0.489)	−0.110 (0.220)

**Note:** ^a^Correlation is significant at the 0.01 level (2-tailed), *P* value derived from Pearson’s and Spearman’s correlation coefficient

## Discussion

A total of 252 study subjects (126 Hypertensive and 126 healthy controls) were involved in this study to compare some hematological parameters among hypertensive and normotensive individuals. The mean age of hypertensive and control individuals were 50.3 ± 11 and 49.8 ± 11.6 years, respectively. Based on the result, hypertensive groups had significantly higher median (IQR) WBC value 6.9 × 10^3^/μl (3.7 × 10^3^/μl) when compared to apparently healthy normotensive controls 5.2 × 10^3^/μl (2.2 × 10^3^/μl). This finding is in agreement with other similar studies by Babu KR et al. [7] and Al-Muhana et al. [19]. Also, this study showed a significant positive correlation of WBC count with diastolic blood pressure, systolic blood pressure and mean arterial pressure.

There is a causal relationship between vascular function and different hematological disorders [17, 20]. Most hypertensive patient’s exhibit increased blood viscosity compared with healthy controls [14]. There is a decreased RBC deformability which could cause an increased microvascular flow resistance, which may result in haemolysis and organ damage [11]. This haemolysis induces release of Hgb in to the plasma which scavenges nitric oxide and causes endothelial dysfunction [21]. There is also functional alterations and abnormalities of platelets in hypertension which is associated with increased risk of clot formation. Activated and large platelets are produced as a result of endothelial dysfunction. These large and activated platelets produce vasoconstrictors. This enhances narrowing of blood vessels; there by high blood pressure and thrombotic disease [22–25].

The relationship between WBC and hypertension may be explained by an increased concentration of stem cell factor (SCF) in serum [26]. During HTN, there is a vascular endothelial dysfunction [27, 28]. Thus, to repair this dysfunction SCF/c-kit increases. The SCF has an important role in differentiation and proliferation of haematopoietic cells [26, 29]. This pathway might increase WBC via its participation in the differentiation and proliferation of haematopoietic cells. Additionally, white blood cells are inflammatory marker and tends to increase during HTN which is supported by Kim D-J et al. [30]. But in contradiction to this study, a study conducted in São Paulo, Brazil showed lower mean value of WBC count in hypertensive individuals when compared to apparently healthy normotensive subjects. But there was no significant association [31]. This difference may be due to differences in the study subjects. The study subjects included in this study were HTN confirmed but the study subjects in São Paulo, Brazil were without a previous diagnosis of high blood pressure.

Similarly, the current study showed significantly higher median (IQR) RBC value 5 × 10^6^/μl (0.88 × 10^6^/μl) when compared to apparently healthy controls 4.88 × 10^6^/μl (0.58 × 10^6^/μl). This is supported by Babu KR et al. [7], Reis RS et al. [31] and Bruschi G et al. [32]. Also, RBC count showed significantly positive correlation with diastolic blood pressure, systolic blood pressure and mean arterial pressure. The possible mechanisms of the association between RBC and blood pressure are not entirely known but the study showed that it may be associated with stem cell factor. Stem cell factor (SCF)/c-kit signaling proteins are increased in hypertensive individuals [26]. Since it is involved in repairing of damaged blood vessels, the expression of stem cell factor (SCF)/c-kit signaling proteins are relatively high during blood vessel repair. Thus, as a result of SCF, RBC number will be increase via the participation of SCF in the differentiation and proliferation of haematopoietic cells [29].

In the present study, Hgb value was significantly increased in the hypertensive group compared to normotensive groups. This findings is in agreement with supported studies done by Babu KR et al. [7] and Al-Muhana et al. [2, 19] but it contradicts with a study conducted in São Paulo, Brazil [31]. Hgb value has shown a positive correlation with systolic, diastolic and mean arterial pressure in hypertensive groups which is similar to the study conducted by Atsma F et al. in France [33].

The association between HTN and Hgb level may be explained by Hgb and arginase enzyme effects on nitric oxide (NO) bioavailability [21, 34]. During HTN, there is a possibility of hemolysis. But, whether hemolysis is a cause or effect of hypertension remains unclear. Most studies suggest that hypertension is a complication of hemolysis and associated with hemolytic anemia [35]. In addition to this, blood disorders such as polycythemia vera and essential thrombocythemia, causes hypertension [20]. Polycythemia vera will cause an increase in relative red cell mass and whole blood viscosity, and thereby increase peripheral resistance to blood flow. If there is peripheral resistance in the microcirculation, there will be a possibility of hemolysis [21]. During hemolysis, hemoglobin and arginase enzyme are released in to circulation from erythrocytes. This free Hgb is scavenger of nitric oxide which is produced in the endothelial cell that lines the blood vessels and important for relaxation of blood vessels. On the other hand, arginase enzyme depletes the substrate used for NO synthesis by conversion of arginine to ornithine, thus reducing NO production. This conditions leading to endothelial dysfunction and ultimately activation of platelets and clots [21, 36, 37]. Therefore, if free Hgb scavenges nitric oxide and arginase enzyme depletes substrates used for NO production, blood vessel dilation decreases, which in turn causes increased blood pressure.

In our study, the median (IQR) value of HCT significantly increased in hypertensive individuals compare to normotensive individuals. These findings are also familiar to Babu KR et al. [7]. In bivariate correlation analysis, HCT value has shown a positive correlation with systolic, diastolic and MAP blood pressure in hypertensive groups. The reasonable mechanisms underlying the association between HCT and blood pressure is that HCT is a determinant factor for high whole blood viscosity during hypertension. This may lead to a peripheral resistance to blood flow and high blood pressure [7, 11]. The evidence showed that, most hypertensive patients exhibit increased blood viscosity compared with healthy controls [14]. Therefore, high hematocrit in hypertension could reflect a true increase in red blood cell mass as well as hemo-concentration caused by a reduction in plasma volume. In contrary to aforementioned result, contradicted study conducted at University of Port Harcourt teaching hospital, Nigeria [38] and Saudi Arabia [19] reported that HCT was not significantly differ between hypertensive patients and normotensive individuals. This difference may be due to difference in sample size.

In our study, RDW increased significantly in hypertensive groups compared to normotensive individuals. Most studies suggest that higher RDW, which is a measure of the variability in the circulating erythrocytes’ size, may be resulted from ineffective erythropoiesis due to chronic inflammation during hypertension [39, 40].

In this study MCV and MCHC were increased significantly in hypertensive groups but there were no significant differences in MCH. But other studies in these parameters showed contradicted ideas. For example a study conducted by Babu KR et al. [7] showed significantly lower MCV, significantly higher MCHC and higher but no difference MCH value. In São Paulo, Brazil, MCV were similar [31], in France MCV is lower by 2% [32] and a study in Saudi Arabia showed no significant differences of MCV, MCH and MCHC [19].

In the present study, the median (IQR) value of PLT count, mean value of MPV and PDW were increased in hypertensive groups than controls. Even though statistically not significant, median value of PLT count was slightly higher in hypertensive groups. The possible explanation for this could be related to consumption of platelets. During hypertension, there is endothelial dysfunction and this leads to platelet activation and clot formation. Then platelets will be consumed and there number does not increase as expected [25, 41, 42]. However, statistically significant increment of MPV and PDW were found in hypertensive groups compared to normotensive groups. This finding is in accordance with the previous findings by Babu et al. [7], Al-Muhana et al. [19], Bruschi et al. [32] and Ates et al. [43].

In our study, PLT count positively correlated with blood pressure indices. The possible mechanisms might be related to vascular complication in hypertensive groups. High blood pressure causes endothelial damage via shear stress, which results in an increase in platelet activation [43]. When platelet production is induced, there could be increment in platelet count, MPV and PDW [44]. Evidence suggests that PLT consumption increase at the site of injured blood vessel. During this condition larger PLTs would be released from the bone marrow because larger PLTs are hemostatically more active than mature PLT. Because larger PLTs are hemostatically more active, the presence of larger PLTs is probably a risk factor for developing coronary thrombosis and myocardial infraction [41, 42].

Since antihypertensive therapy reduces blood pressure and improve endothelial function, their effect didn’t assess in this study. Additionally, cell free hemoglobin analysis was not considered. Therefore, further cohort study is required.

## Conclusion

In the present study, the median (IQR) value of WBC, RBC, Hgb, HCT, MCV and the mean value of MCHC, RDW, MPV and PDW were significantly higher in the hypertensive group compared to apparently healthy controls. But platelet count and MCH showed no statistically significant difference between hypertensive and normotensive groups. In this study there was statistically significant positive correlations of WBC, RBC, Hgb, HCT and PLT with blood pressure indices (diastolic blood pressure, systolic blood pressure and mean arterial pressure) among hypertensive individuals. Impaired hematological parameters may strongly indicate hypertensive end-organ damage. Hematological complication such as RBCs reduction in deformability and an increase in the size causes hemolysis, high Hgb and low levels are associated with cardiovascular risk and activation of platelets is risk factor for thrombotic diseases observed during hypertension. Therefore, it is important to assess changes in hematological parameters for hypertensive patients which may help to prevent such complications associated hematological disorders.

## Additional files

Additional file 1: Questionnaire. Data collection questionnaire designed for the comparative cross-sectional study of some hematological parameters of hypertensive and normotensive individuals at the university of Gondar hospital, Northwest Ethiopia. (DOCX 15 kb)

Additional file 2: Laboratory result registration form. Laboratory result registration form designed for the registration of hematological parameter results of hypertensive patients and normotensive controls at university of Gondar Hospital, Northwest Ethiopia. (DOCX 13 kb)

## Abbreviations

BP: Blood pressure
CBC: Complete blood cell count
DBP: Diastolic blood pressure
EDTA: Ethylene diamine tetra-acetate
HCT: Hematocrit
Hgb: Hemoglobin
HTN: Hypertension
MAP: Mean arterial pressure
MCH: Mean cell hemoglobin
MCHC: Mean cell hemoglobin concentration
MCV: Mean cell volume
MI: Myocardial infarction
MmHg: Millimeters mercury
MPV: Mean platelet volume
NO: Nitric oxide
PLT: Platelets
RBC: Red blood cells
RDW: Red blood cell distribution width
SBP: Systolic blood pressure
SCF: Stem cell factor
WBC: White blood cells
