# Supplementary material for: A comparative cross-sectional study of some hematological parameters of hypertensive and normotensive individuals at the university of Gondar hospital, Northwest Ethiopia
Source: BMC Hematol. 2017 Nov 28;17:21. doi: 10.1186/s12878-017-0093-9 (PMC5704458; doi:10.1186/s12878-017-0093-9)
Supplement: Supplementary file 1 — Questionnaire. Data collection questionnaire designed for the comparative cross-sectional study of some hematological parameters of hypertensive and normotensive individuals at the university of Gondar hospital, Northwest Ethiopia. (DOCX 15 kb) [file 12878_2017_93_MOESM1_ESM.docx]

**Questionnaire**

Data collection questionnaire designed for the comparative cross-sectional study of some hematological parameters of hypertensive and normotensive individuals at the university of Gondar hospital, Northwest Ethiopia

**Code: _________**

| **Sr. No.** | **Questions** | **Possible Responses** |
| --- | --- | --- |
|  | Age (in years) | __________ |
|  | Sex | 1. Male 2. Female |
|  | Residency | 1. Urban 2. Rural |
|  | Weight: | _____kg |
|  | Height: | ______cm |
|  | BMI (By calculation as: Wight in Kg/Height (cm)^2^) | ______ |
|  | Type of hypertension | 1. Primary hypertension 2. Secondary hypertension |
|  | Duration of hypertension since diagnosis (years) | _______ |
|  | Blood pressure | Systolic ______mmHg  Diastolic______ mmHg  Mean Arterial Pressure: ___mmHg |
|  | Alcohol consumption | 1.Yes 2. No |
|  | Smoking | 1.Yes 2. No |
|  | Medical history of any of diseased conditions like diabetes mellitus, cardiac disease, kidney and liver diseases, etc.. | 1.Yes 2. No |
|  | History of infectious diseases signs or symptoms | 1.Yes 2. No |
|  | Taking of antibiotics, iron, vitamin B12 or folate supplementations or any other medications | 1.Yes 2. No |
