# Supplementary material for: A comparative cross-sectional study of some hematological parameters of hypertensive and normotensive individuals at the university of Gondar hospital, Northwest Ethiopia
Source: BMC Hematol. 2017 Nov 28;17:21. doi: 10.1186/s12878-017-0093-9 (PMC5704458; doi:10.1186/s12878-017-0093-9)
Supplement: Supplementary file 2 — Laboratory result registration form. Laboratory result registration form designed for the registration of hematological parameter results of hypertensive patients and normotensive controls at university of Gondar Hospital, Northwest Ethiopia. (DOCX 13 kb) [file 12878_2017_93_MOESM2_ESM.docx]

**Laboratory result registration form**

Laboratory result registration form designed for the registration of hematological parameter results of hypertensive patients and normotensive controls at university of Gondar Hospital, Northwest Ethiopia

| Code | Age | Sex | WBC (10^3^/μl) | RBC  (10^6^/μl) | Hgb  (g/dl) | HCT  (%) | Platelet  (10^3^/μl) | MCV (fl) | MCH (Pg) | MCHC (%) | RDW (fl) | MPV (fl) | PDW (fl) |
| --- | --- | --- | --- | --- | --- | --- | --- | --- | --- | --- | --- | --- | --- |
|  |  |  |  |  |  |  |  |  |  |  |  |  |  |
|  |  |  |  |  |  |  |  |  |  |  |  |  |  |
|  |  |  |  |  |  |  |  |  |  |  |  |  |  |
|  |  |  |  |  |  |  |  |  |  |  |  |  |  |
|  |  |  |  |  |  |  |  |  |  |  |  |  |  |
|  |  |  |  |  |  |  |  |  |  |  |  |  |  |
|  |  |  |  |  |  |  |  |  |  |  |  |  |  |
|  |  |  |  |  |  |  |  |  |  |  |  |  |  |
|  |  |  |  |  |  |  |  |  |  |  |  |  |  |
|  |  |  |  |  |  |  |  |  |  |  |  |  |  |
|  |  |  |  |  |  |  |  |  |  |  |  |  |  |
|  |  |  |  |  |  |  |  |  |  |  |  |  |  |
|  |  |  |  |  |  |  |  |  |  |  |  |  |  |
|  |  |  |  |  |  |  |  |  |  |  |  |  |  |
|  |  |  |  |  |  |  |  |  |  |  |  |  |  |
|  |  |  |  |  |  |  |  |  |  |  |  |  |  |
|  |  |  |  |  |  |  |  |  |  |  |  |  |  |
|  |  |  |  |  |  |  |  |  |  |  |  |  |  |
|  |  |  |  |  |  |  |  |  |  |  |  |  |  |
|  |  |  |  |  |  |  |  |  |  |  |  |  |  |
|  |  |  |  |  |  |  |  |  |  |  |  |  |  |
